# Severe Intraoperative Anaphylaxis Related to Thymoglobulin during Living Donor Kidney Transplantation

**DOI:** 10.3390/antib9030043

**Published:** 2020-08-18

**Authors:** Muhammad I. Saeed, Ryan D. Nicklas, Vikas Kumar, Rajan Kapoor, Imran Y. Gani

**Affiliations:** 1 Department of Surgery, Augusta University Medical Center, Augusta, GA 30912, USA; 2 Department of Anesthesiology, Augusta University Medical Center, Augusta, GA 30912, USA; Rnicklas@augusta.edu (R.D.N.); Vikkumar@augusta.edu (V.K.); 3 Department of Nephrology, Hypertension and Transplant Medicine, Augusta University Medical Center, Augusta, GA 30912, USA; Rkapoor@augusta.edu (R.K.); Igani@augusta.edu (I.Y.G.)

**Keywords:** living donor kidney transplantation, rabbit anti-thymocyte globulin, anaphylactic shock

## Abstract

Anaphylaxis secondary to thymoglobulin (anti-thymocyte globulin) is a rare condition that can be life threatening. Thymoglobulin is a rabbit-derived T-cell depleting polyclonal immunoglobulin. It is commonly used for induction immunosuppression and/or for treatment of acute rejection in renal transplantation. We report a case of a living kidney transplant recipient who developed intraoperative anaphylactic shock secondary to thymoglobulin. The patient had a history of pet rabbit exposure. This case report highlights the importance of prompt identification and management of intraoperative anaphylaxis, which is key to a successful outcome. Induction immunosuppression selection based on patient characteristics is important. Communication between the anesthesia team and surgeons played a key role in stopping the donor surgery.

## 1. Introduction

Thymoglobulin (anti-thymocyte globulin) is a rabbit-derived purified polyclonal immunoglobulin directed against human T-lymphocytes. Thymoglobulin is commonly used for induction immunosuppression and/or for treatment of acute rejection in renal transplantation [1]. Anaphylaxis to thymoglobulin is rare; however, prior exposure to rabbit antigens may predispose patients to developing an anamnestic response leading to an anaphylactic reaction [2]. Intraoperative anaphylactic reactions present a great challenge to the anesthesiologist, since they can rapidly become life threatening. We present a case of serious anaphylactic shock following thymoglobulin administration during a living-donor renal transplantation in a patient with prior exposure to rabbits.

## 2. Case Presentation

**Figure 1:**
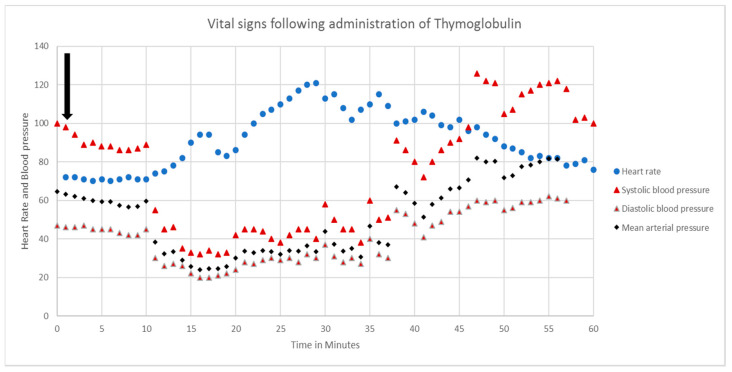
Intraoperative vital signs after administration of thymoglobulin.

The patient is a 67-year-old Caucasian female with end-stage renal disease who developed anaphylactic shock following thymoglobulin infusion during living-donor kidney transplant surgery. She had a smooth and uneventful induction and maintenance of anesthesia. She was started on 75 mg of thymoglobulin intravenous (IV) infusion intraoperatively after pre-medications, which included diphenhydramine 25 mg IV, acetaminophen 650 mg via nasogastric tube, methylprednisolone 500 mg IV and famotidine 20 mg IV. Within minutes of initiation of thymoglobulin infusion, the patient experienced profound hypotension with systolic blood pressures (SBP) ranging between 40 and 60 mmHg, mean arterial pressures (MAP) ranging from 30 to 40 mmHg, tachycardia (heart rate 110–120) and respiratory acidosis (ABG drawn at the time: pH 7.18/pO_2_ 146/pCO_2_ 61/HCO_3_ 23.4/BE-5.4) Figure 1.

Due to suspected anaphylaxis, the thymoglobulin infusion was stopped immediately. Although the airway was intact with a good endotracheal tube position, there was bilateral wheezing and difficulty in ventilation. Her peak airway pressures went up and ranged between 35 and 40 cm H_2_O. An intraoperative trans-esophageal echocardiogram was not suggestive of pulmonary embolism (no right ventricle dysfunction) or myocardial infarction, and showed a hyperdynamic left ventricle. Ultrasound of the chest showed no evidence of pneumothoraces. There was no evidence of acute hemorrhage either. The patient had no documented latex allergy. The patient’s hypotension was resistant to crystalloid and colloid fluid boluses, and required multiple vasopressors (epinephrine norepinephrine and vasopressin) to maintain blood pressure. We aborted the kidney transplant procedure and updated family members about the intraoperative events. The patient remained intubated and was transported to the surgical intensive care unit. She was maintained on high doses of vasopressors, ventilator support and other supportive measures to optimize hemodynamic status. The patient received high-dose intravenous steroids. The patient also underwent one session of plasmapheresis, after which she developed retroperitoneal hematoma requiring re-exploration that showed generalized oozing with no active hemorrhage. The patient was able to be weaned off vasopressors within 48 h of the event. 72 h after the initial event and with basiliximab induction, the patient successfully received the living-kidney transplant. Subsequent discussion with the patient revealed significant exposure to rabbits as pets. Her intraoperative immunologic studies were significant for elevated tryptase and histamine. She also had elevated anti-rabbit protein IgE levels (Table 1).

## 3. Discussion

Thymoglobulin infusion is usually tolerated well without significant complications if patients receive pre-medications, and the medication itself is infused slowly to avoid cytokine release syndrome. The potential infusion-related side effects include flu-like symptoms such as fever, chills, dyspnea, nausea, vomiting and diarrhea [1]. There are very few instances of thymoglobulin anaphylaxis reports in the medical literature [2,3,4,5]. Brabant et al. report a patient with a history of atopy and hypersensitivity reactions on exposure to rabbits [2]. In our case, the patient had a pet rabbit, but denied having common allergic symptoms upon exposure, including rhinitis, rash, wheezing or angioedema.

Intraoperative anaphylactic shock can be a life-threatening situation with a mortality of 3–10%, and an additional 2% can have severe neurological damage [6]. The incidence of anaphylaxis during general anesthesia has been reported to range from 1 in 4000 to 1 in 25,000 cases [7,8]. Prompt diagnosis and management are required for favorable outcomes. Anaphylaxis is a serious systemic IgE-mediated allergic reaction. Anaphylaxis after general anesthesia induction can occur due to medications, e.g., thiopental, neuromuscular blocking agents like succinylcholine and rocuronium, opioids, protamine, methylene blue and preoperative antibiotics [7,8]. In transplant cases, thymoglobulin should also be highly considered as a causative agent. In our case, the induction and maintenance of anesthesia was uncomplicated, and shock occurred within a few minutes of starting thymoglobulin infusion. The quick onset and severity of shock in our case, along with the supporting laboratory data, argue in favor of an IgE-mediated process. Other causes of intraoperative anaphylaxis include latex allergy, blood transfusion and bone cement [8]. We did not administer any blood products and the patient denied any latex allergy prior to surgery. Roncati et al. reported a very rare case of anaphylaxis due to thymoglobulin carbohydrate excipient [6].

The signs and symptoms of anaphylaxis can occur within minutes of exposure to an offending agent; however, some reactions may develop more than 30 min later [8]. Late-phase or biphasic reactions can occur 8–12 h after initial exposure, and protracted/severe anaphylaxis might last up to 32 h, despite aggressive management [8]. The clinical signs and symptoms of intraoperative anaphylaxis include profound hypotension, tachycardia, difficulty in lung inflation, oxygen desaturation and a decrease in end-tidal carbon dioxide (ETCO_2_). Difficulty ventilating secondary to bronchospasm can be the first sign of anaphylaxis intraoperatively in patients who have undergone general anesthesia. It can also be the sole manifestation of anaphylaxis in an intubated patient [9]. Hypotension is secondary to increased vascular permeability, which can lead to as much as 50% of the intravascular volume leaking into extravascular space within minutes [8]. This concept is very important to understand in the management of anaphylactic patients as hemodynamic collapse might occur rapidly with no cutaneous or respiratory symptoms [8,9]. Further, one can easily miss the cutaneous manifestations of anaphylaxis, such as rash or flushing, due to the patient being under surgical drapes. Kroigaard et al. describe the classification of intraoperative anaphylaxis manifestations [7,10].

It is important to ask for help when there is a high suspicion for anaphylaxis. The anesthesia provider should quickly notify the surgeon regarding the patient’s clinical condition and patient stabilization should be the top priority. The anesthesia provider and surgical staff should immediately request additional personnel to help manage these rapidly evolving situations, wherein multiple processes happen simultaneously [7]. It is important to rule out other causes of acute cardiovascular collapse, including myocardial infarction, tension pneumothorax, pulmonary embolism, hereditary angioedema and seizure.

The management of anaphylaxis starts from securing the airway for oxygenation and the initiation of rapid fluid resuscitation. Epinephrine injection is the first line treatment option [7,8,11]. Adults should also receive normal saline 5–10 mL/kg in the first 5 min followed by maintenance fluids [8]. The next step should be to administer colloid solutions if hypotension persists. Diphenhydramine 1 to 2 mg/kg or 25 to 50 mg IV should be considered as a second line therapy to epinephrine. Combination of diphenhydramine with ranitidine is superior to diphenhydramine alone [8]. Beta-agonist (e.g., albuterol) nebulization should be considered to decrease bronchospasm, and repeated as necessary. FiO_2_ and PEEP are adjusted to maintain adequate oxygenation. In patients refractory to epinephrine and fluids, blood pressure is supported with additional vasopressors. Glucocorticoids should not be considered as a first line therapy, but can be helpful in preventing protracted anaphylaxis. They can be administered every 6 h [8,12]. The primary role of glucocorticoids is to prevent the second phase response, as described by Ellis et al. [13]. Glucagon infusions can be considered in patients who are on beta-blockers pre-operatively, as epinephrine might be ineffective in such cases [8,10,13]. There is no literature evidence to support the use of plasmapheresis for anaphylaxis conditions. However, in this case, the presumed cause of anaphylaxis was thymoglobulin. Thymoglobulin is an antibody medication, and has been shown to be removed by plasmapheresis [14].

Laboratory work up at the time or immediately after the event is important in supporting the diagnosis. Lieberman et al. recommended a list of tests to determine the cause of anaphylaxis [8]. The anaphylactic origin and IgE-mediated pathogenesis of shock in our patient is strongly supported by elevated levels of tryptase, histamine and anti-rabbit protein IgE. The timing of laboratory tests is important, as plasma histamine levels begin to increase 5–10 min after symptom onset and remain elevated for 30–60 min [8,11]. Urinary methyl-histamine levels are elevated for a longer duration of time, up to 24 h [8]. Serum tryptase levels peak one to one-and-a-half hours after the onset of symptoms, and can persist for as long as five to six hours [8,15]. Blood samples obtained at the time of anaphylactic reaction can be used to measure allergen-specific IgE levels based on the patient’s history.

Preoperatively, if a rabbit allergy is suspected or there is a known history of significant rabbit exposure (e.g., pets, veterinary personnel, frequent consumption of rabbit meat), then preoperative hypersensitivity testing, and potentially a change in induction immunosuppression, should be considered [5]. Skin prick testing (SPT) for thymoglobulin is limited by its availability and cost. Discussions with the patient regarding risks and benefits of testing, desensitization to anti-thymocyte globulin, and the availability of rescue medications and necessary equipment should be held. Millar et al. reported that desensitization can be difficult and should be approached with caution [16]. We suggest using a different induction agent in such a situation.

In a dual patient surgical procedure, communication between recipient and donor surgeons is also important in order to halt the donor surgery immediately. In this case, the donor surgery was halted just prior to explantation of the already dissected kidney. The donor was extubated and kept in the hospital on bedrest with heparin anticoagulation and sequential compression devices for 72 h. After the recipient was hemodynamically stable, the living donor kidney transplant was successfully performed.

Post-transplant immunosuppression medication compliance and regular follow up are important after an episode of thymoglobulin allergy, as treatment options for kidney transplant rejection are limited in such situations. In our case, we added thymoglobulin to the patient’s allergy medication list.

## 4. Conclusions

This case demonstrates the importance of prompt identification and management of intraoperative anaphylaxis. Communication between medical providers to facilitate appropriate management is of the utmost importance. The induction immunosuppression agent should be carefully selected in patients with significant previous rabbit exposure or allergy.

## Figures and Tables

**Table 1:** Test Results.

Test	Patient’s Result	Reference Range
Anti-rabbit protein IgE	1.44 kU/L	<0.35 kU/L
Serum Tryptase	36.9 ng/mL	<15 ng/mL
Serum Histamine	107 ng/mL	<65 ng/mL
